# Characterization of pyruvate metabolism and citric acid cycle patterns predicts response to immunotherapeutic and ferroptosis in gastric cancer

**DOI:** 10.1186/s12935-022-02739-z

**Published:** 2022-10-13

**Authors:** Xu Wang, Bing Xu, Jing Du, Jun Xia, Guojie Lei, Chaoting Zhou, Jiayu Hu, Yinhao Zhang, Sufeng Chen, Fangchun Shao, Jiyun Yang, Yanchun Li

**Affiliations:** 1 Sichuan Provincial Key Laboratory for Human Disease Gene Study, Center for Medical Genetics, Sichuan Academy of Medical Sciences & Sichuan Provincial People’s Hospital, University of Electronic Science and Technology, Chengdu, 610072 Sichuan China; 2 Laboratory Medicine Center, Department of Clinical Laboratory, Zhejiang Provincial People’s Hospital (Affiliated People’s Hospital, Hangzhou Medical College), Hangzhou, 310014 Zhejiang China; 3 Department of Clinical Laboratory, Hangzhou Women’s Hospital, Hangzhou, 310005 Zhejiang China; 4 Department of Pulmonary and Critical Care Medicine, Zhejiang Provincial People’s Hospital (Affiliated People’s Hospital, Hangzhou Medical College), Hangzhou, 310014 Zhejiang China; 5 Department of Central Laboratory, Affiliated Hangzhou first people’s Hospital, Zhejiang University School of Medicine, Hangzhou, 310006 Zhejiang China

**Keywords:** Gastric cancer, Pyruvate metabolism, Citric acid cycle, Tumor microenvironment, Tumor mutational burden, Immunotherapy

## Abstract

**Background:**

Gastric cancer is one of the most common malignancies of the digestive system with a high lethal rate. Studies have shown that inherited and acquired mutations in pyruvate metabolism and citric acid cycle (P-CA) enzymes are involved in tumorigenesis and tumor development. However, it is unclear how different P-CA patterns affect the tumor microenvironment (TME), which is critical for cancer progression.

**Methods:**

This study mainly concentrated on investigating the role of the P-CA patterns in multicellular immune cell infiltration of GC TME. First, the expression levels of P-CA regulators were profiled in GC samples from The Cancer Genome Atlas and Gene Expression Omnibus cohorts to construct a consensus clustering analysis and identify three distinct P-CA clusters. GSVA was conducted to reveal the different biological processes in three P-CA clusters. Subsequently, 1127 cluster-related differentially expressed genes were identified, and prognostic-related genes were screened using univariate Cox regression analysis. A scoring system was then set up to quantify the P-CA gene signature and further evaluate the response of the patients to the immunotherapy.

**Results:**

We found that GC patients in the high P-CA score group had a higher tumor mutational burden, higher microsatellite instability, and better prognosis. The opposite was observed in the low P-CA score group. Interestingly, we demonstrated P-CA gene cluster could predict the sensitivity to immunotherapy and ferroptosis-induced therapy.

**Conclusion:**

Collectively, the P-CA gene signature in this study exhibits potential roles in the tumor microenvironment and predicts the response to immunotherapeutic. The identification of these P-CA patterns may significantly accelerate the strategic development of immunotherapy for GC.

**Supplementary Information:**

The online version contains supplementary material available at 10.1186/s12935-022-02739-z.

## Introduction

Gastric cancer (GC) originates from the mucosal epithelium of the stomach. A higher incidence has been reported in people aged > 50 years, the ratio of men to women is about 2:1 [1, 2]. In response to changes in diet structure, work stress, and chronic infection with *Helicobacter pylori*, the incidence of GC is increasing and has shown a younger trend in recent years [3, 4]. Despite substantial improvements in its diagnosis and treatment, including curative surgical resection and adjuvant therapy, outcomes of GC remain poor, and the overall survival is less than 40% [5–8]. Therefore, in order to achieve greater efficacy and improve the prognosis for GC patients, we developed a prognostic model through the identification of novel P-CA patterns to provide insights into personalized treatment for GC patients.

Pyruvate metabolism and the citric acid cycle (P-CA) are regarded as fundamental units for generating energy and biosynthetic precursors during cell growth and proliferation. Upon uptaken into cells, glucose is initially converted into pyruvate in the cytoplasm, and the full oxidation of glucose occurs in the mitochondria through the citric acid cycle under aerobic conditions [9]. Unlike aerobic metabolism, pyruvate is metabolized to lactate in the cytoplasm under anaerobic conditions [10]. Notably, cancer cells tend to metabolize glucose to lactate in aerobic environments, which is known as the “Warburg effect” [11], allowing for enhanced glucose uptake and utilization by tumor cells. Research showed that mutations in the citric acid cycle enzymes contribute to the dysregulation of metabolites and are associated with oncogenesis [12]. Isocitrate dehydrogenase (IDH) is a key member of metabolic enzymes involved in the oxidative decarboxylation of isocitrate to α-ketoglutaric acid (α-KG) and the reduction of NADH to NADPH [13]. Mutations or aberrant expressions of IDH have been documented in numerous types of cancers [14–16]. Succinate dehydrogenase (SDH) is an enzyme localized in the mitochondria that plays a catalytic role in the oxidation of succinate, which subsequently produces fumarate. It also functions as a tumor suppressor by reducing succinate and inactivating hypoxia-inducible factor 1α [17]. Mutations in SDH can act as promoting factors for the occurrence and development of diseases, including tumorigenesis and genomic instability [18]. A previous study reported that miR-422a modulates the metabolism and malignancy of GC cells by targeting pyruvate dehydrogenase kinase 2 [19]. In addition, the accumulated intermediate products of P-CA contribute to cancer progression by activating or inhibiting specific signaling pathways, thereby influencing metabolic pathways [12].

Tumors are highly heterogeneous tissues surrounded by the tumor microenvironment (TME), which is substantially different from a healthy microenvironment, including metabolism, biosynthetic processes, and physical-chemical environment [20]. Immunotherapy is an emerging and representative therapeutic modality for cancer treatment [21, 22]. The types of immunotherapy include immune checkpoint blockade (ICB) antibody or immune checkpoint inhibitor (ICI) treatment, chimeric antigen receptor T-cell therapy, and cancer vaccines [22], among which, immunotherapy targeting immune checkpoints have achieved significant benefits in a wide variety of malignancies [23]. The response of tumor cells to immunotherapy agents and patient survival rate are tightly linked to the TME [24]. As reported, tumor-derived lactate strongly inhibits the proliferation of natural killer (NK) cells and T lymphocytes and limits their anticancer immunity [25, 26]. Interactions between the TME and tumor cells are derived in part from diffusible metabolites [27]. Targeting metabolic pathways in cancer cells might pave pathways for a breakthrough in anticancer treatment in the future. Therefore, it is crucial to understand the type of tumor-infiltrating immune cells influenced by P-CA patterns for cancer therapy.

In this study, three gene clusters were proposed in GC patients depending on the different expression levels of P-CA-related genes. A scoring system was then set up to quantify the P-CA pattern and further evaluate the response of the patients to immunotherapy. It was elucidated that P-CA acts as a vital player in the development of GC via regulating the TME and can be a parameter to increase the predictability of the response to immunotherapy.

## Materials and methods

### Gastric cancer dataset acquisition and preprocessing

Processed FPKM gene expression data, genome mutation data, and clinicopathologic information of 375 gastric cancer patients and 32 normal control were obtained from The Cancer Genome Atlas (TCGA, available online: https://portal.gdc.cancer.gov/). The data set GSE84437 (433 tumor samples) were derived from Gene Expression Omnibus (GEO, available online: https://www.ncbi.nlm.nih.gov/geo/). Patients without prognostic information were excluded from the study. Before data analysis, the RNA-Seq FPKM expression values were transformed into transcripts per kilobase million (TPM) values using R software.

### Consensus clustering of P-CA

49 genes in P-CA were acquired. Independent prognostic genes were obtained after performing univariate Cox regression analyses. The identification of P-CA clusters was conducted with unsupervised cluster analysis through the R package “ConsensusClusterPlus” on basis of the expression profiles of these genes.

### Evaluation of the relationship between clinical characteristics and different P-CA clusters

To determine the relationships between P-CA clusters and clinical phenotypes, clinical information of GC including age, sex, TNM stage, and survival status were summarized for analyses of the association with P-CA clusters, the result was visualized in a heatmap. Kaplan Meier curves were used to determine survival prognostic differences in GC among the three clusters with the “survival” and “survminer” R packages.

### Gene set variation analysis and gene enrichment function annotation

GSVA, a nonparametric, unsupervised method for calculating gene set enrichment through expression profiles, was performed to disclose the difference in the activities of P-CA clusters in biological processes. The gene ontology (GO) function annotations of genes were analyzed using the“clusterProfiler” package.

### Immune cell difference analysis

Single sample Gene Set Enrichment Analysis (ssGSEA) was performed to quantify the immune cell infiltration levels, including activated CD8^+^ T cells, activated dendritic cells, giant natural killer T cells, and regulatory T cells. Adjusted *p*-value < 0.05 was considered statistically significant.

### Construction of a P-CA-related prognostic model

Limma package was utilized to survey the common differentially expressed genes (DEGs) among three P-CA clusters. Gene expression was quantified by Transcript per Million (TPM). The intersecting DEGs of the three clusters were obtained for model construction. The gene function annotation and identification of corresponding enriched pathways were performed using the GO and KEGG database based on P-CA-related DEGs with the “clusterProfifiler” package in R and under the condition FDR < 0.01. Principal component analysis based on selected genes was carried out to verify the ability to differentiate clusters. Furthermore, DEGs were analyzed by univariate Cox regression analysis to filtrate the genes related to prognosis and served as input for model training. A scoring system noted as “Pyruvate metabolism and Citric Acid cycle score” (P-CA score) was established based on principal component analysis to quantify the P-CA pattern of individual GC patients. Patients were then alternatively separated into two groups (the high-score group or low-score group) by the maximally selected rank statistics.

### Correlation between the P-CA score and immune-related functions

Group comparisons of P-CA score were performed using a series of analyses, including Kaplan–Meier curves analysis (log-rank tests, *p* < 0.001) to investigate the prognostic value of P-CA score. Meanwhile, stratification analyses on tumor mutation burden were subsequently conducted to assess the predictive ability of the score model. In addition, ssGSEA was used to determine the abundances of immune cell infiltration and Wilcoxon ranked-sum test was used to compare the differential expression of immune checkpoints including PD1, and PDL1 between the two P-CA score groups. Furthermore, the association between the P-CA score and the status of microsatellite instability was also evaluated through correlation analysis.

### Hub genes expression datasets

The validation datasets (GSE27342) were downloaded from the GEO database (http://www.ncbi.nlm.nih.gov/geo/) for further validation. In addition, The gene expression data for GC and the paired adjacent non-cancerous tissues were obtained from The Human Protein Atlas database (HPA, https://www.proteinatlas.org/), and the corresponding immunohistochemistry pictures were taken to show the expression of the target proteins.

### Cell culture

Human gastric cancer cells, BGC823, were preserved and passaged in our laboratory and cultured using DMEM medium (Hyclone, Logan, UT, USA) supplemented with 10% fetal bovine serum (Gibco, Grand Island, NY, USA), penicillin (100 U/mL) and streptomycin (100 µg/mL, Solarbio, China). The plates were placed in a CO_2_ incubator in which the gas composition was 95 vol% air and 5 vol% CO_2_.

### CCK8 assay

Cell viability evaluation was executed by the CCK8 experiment. Cells were seeded in 96-well culture plates (Nest, Biotechnology) at a density of 2 × 10^4^ cells /well. A series of agents including C968 (an allosteric inhibitor of glutamine oxidation pathway), Complex I inhibitor rotenone (Rot), Complex II inhibitor diethyl butylmalonate (DBM), Complex III inhibitor antimycin A (Anti A), and Complex IV inhibitor NaN3 were used in subsequent experiments. A dose of 10µM C968, 10 µM Rot, 2mM DBM, 2.5 µM Anti A, and 15mM NaN3 was administered in combination with erastin to cells for 36 h at 37 °C. Then the supernatant was replaced with CCK8-containing medium for additional 2 h and assayed for cell viability by measuring the absorbance at 450 nm. Each experiment was repeated three times.

### Fluorescent probes staining

BGC823 cells (700,000 per well) were plated in 6-well plates with indicated treatments. The culture solution was discarded, and replenished with culture medium containing 4 µM BODIPY, a fluorescent probe used for detecting the level of cellular lipid peroxides, and incubated for 30 min again. Cells were washed three times with PBS to remove excess BODIPY and subsequently viewed and captured under a confocal microscope.

### Statistical analysis

Correlation coefficients between the TME infiltrating immune cells and the expression level of P-CA regulators were calculated using the spearman and differential expression analyses. Difference comparisons in these groups were performed by one-way analysis of variance (ANOVA) and Kruskal-Wallis test. To obtain the optimum cutoff point for each dataset set according to the relationship between patient survival and P-CA score, the “survminer” R package was carried out. We dichotomized the P-CA scores as low or high by the maximally selected rank statistics. Chi-square tests were used to evaluate the relationships between the P-CA score and the clinical characteristics. The prognostic analysis was implemented using the log-rank test and Kaplan–Meier curves to generate survival curves. Univariate regression analyses were subjected to calculate the hazard ratios (HR) for P-CA regulators and P-CA subtype-related genes. All statistical analyses were performed with R version 4.1.0.

## Results

### The genetic variation landscape of P-CA regulatory factors in gastric cancer

**Fig. 1:**
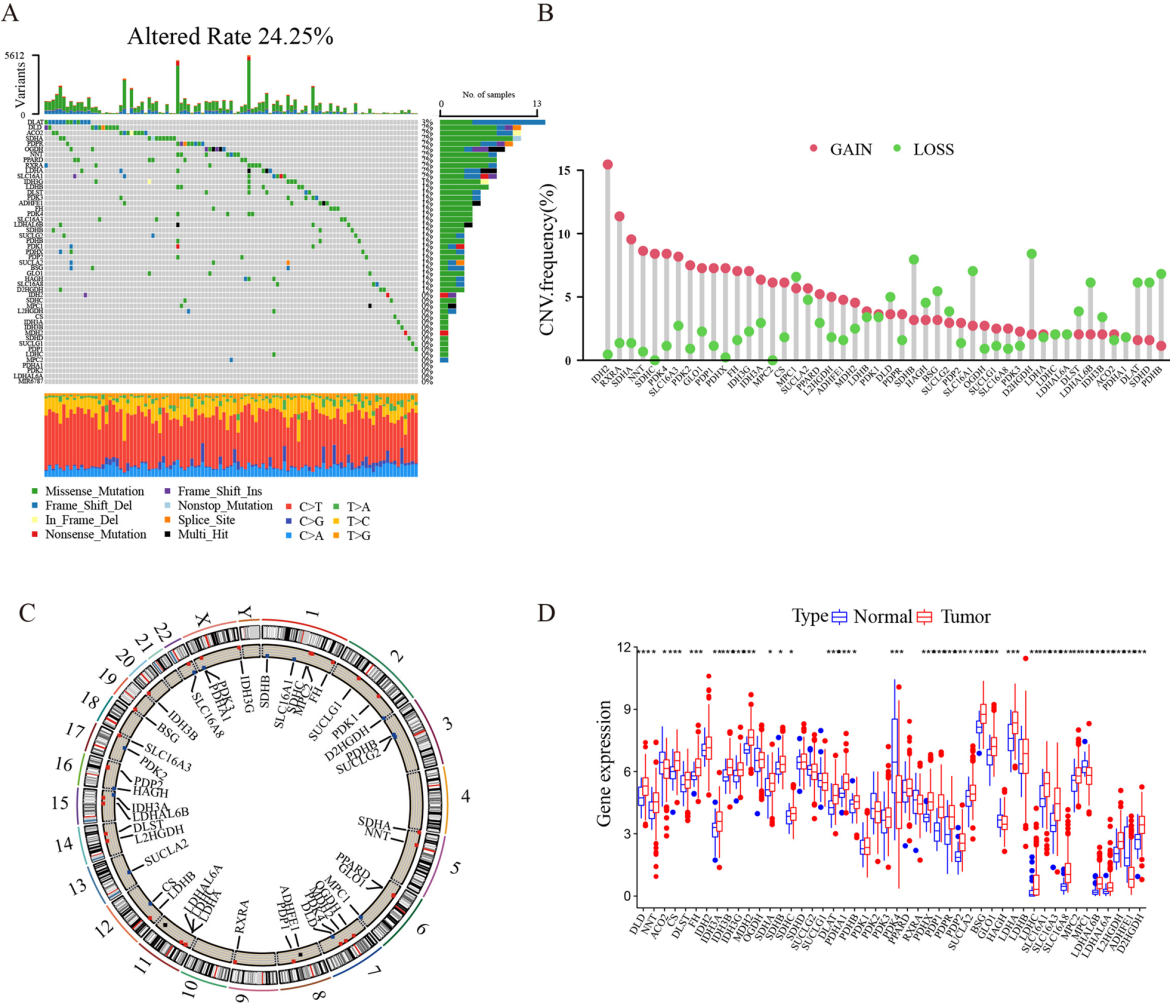
Genetic and expression changes of the P-CA regulators in gastric cancer. **A** Mutation frequency of the P-CA regulators of gastric cancer patients in the TCGA cohort. **B** The CNV alteration frequency of each gene was obtained by statistical analysis of the copy number of P-CA regulators. The abscissa and the ordinate represent the P-CA-related gene and the mutation frequency, respectively. Red indicates an increase in copy number, and green indicates loss of copy number. **C** This circle diagram displayed the alterations sites of P-CA-related genes CNV on 23 chromosomes. **D** Boxplot shows the expression of P-CA regulators between tumor and normal tissues in the TCGA-GC cohort. (*p < 0.05, **p < 0.01, ***p < 0.001, ****p < 0.0001, *p*-value < 0.05 were considered statistically significant). TCGA, The Cancer Genome Atlas; CNV, copy number variation

We firstly identified 49 P-CA regulators in gastric cancer, and the variations in genetic and transcriptomic were investigated. Among the samples downloaded from TCGA-GC mutation dataset, 24.25% carried mutations of 49 P-CA regulatory factors. Among the 49 genes, we observed that *DLAT* exhibited the highest mutation frequency in GC samples. Following this, a slightly lower mutation frequency was found in *ACO2*, *SDHA*, *PDPR*, *OGDH*, *NNT*, *PPARD*, *RXRA*, *LDHA*, and *SLC16A1*, in contrast, there were no mutations in *IDH2, SDHC, MPC1, L2HGDH, CS, IDH3A, IDH3B, MDH2, SDHD, SUCLG1, PDP1, LDHC*, and *MPC2* (Fig. 1A). For that *DLAT* had the highest mutation rate, patients were separated into *DLAT* wild-type group and *DLAT* mutation group. As shown in Additional file 1: Fig. S1, we found that *SLC16A1, FH, SDHB, PDHB, PDK1, L2HGDH, CS*, and *SDHD* expression was higher in the *DLAT* mutation group (Additional file 1: Fig. S1A), whereas the expression level of *PDK4* was increased in *DLAT* wild-type group (Additional file 1: Fig. S1B). The results tentatively proved the functionality of *DLAT* in regulating P-CA gene expression in gastric cancer. Copy number profiling showed that there were 48 of these genes exhibited copy number variation (CNV). For *IDHA2, RXRA, SDHA, NNT, SDHC, PDK4, SLC16A3, PDK2, PDP1, PDHX, FH, IDH3G, IDH3A, MPC2*, and *CS*, their most prevalent status were copy number gain, for *SDHB, SLC16A1, D2HGDH, DLST, LDHAL6B, DLAT, SDHD*, and *PDHB*, the most prevalent status were copy number loss (Fig. 1B). The chromosome position of the CNV mutation of the P-CA regulatory factors were indicated on the circle diagram (Fig. 1C). We conducted an additional analysis to reveal the difference in gene expression of P-CA regulators between normal and tumor samples. Compared with normal tissues, the majority of P-CA modulators were significantly upregulated in GC tissues, regardless of CNV gains or CNV losses (Fig. 1D), indicating that CNV mutations may not be a unique factor in the regulation of P-CA-related genes expression.

### Identification of distinct P-CA clusters based on P-CA regulators

**Fig. 2:**
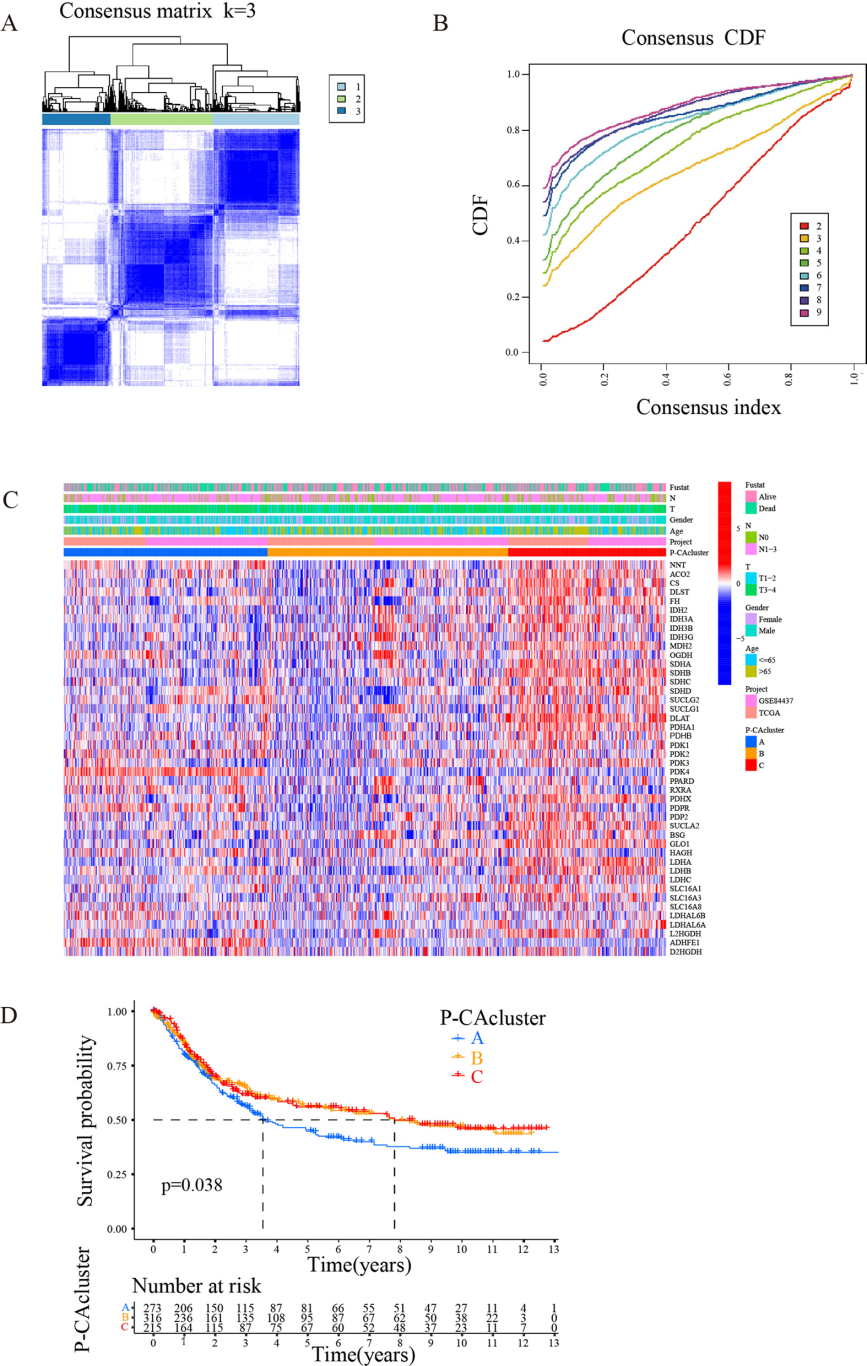
P-CA patterns and the clinicopathological characteristics of each pattern. **A** The consensus matrix heatmap revealed three clusters (k = 3) and their corresponding region. **B** Different curves correspond to given different values of K. **C** Heat map showing the expression patterns of 49 P-CA regulator genes with different clinicopathologic characteristics in the TCGA-GC and GSE84437 cohort. **D** Kaplan–Meier curves of the gastric cancer patients based on P-CA clusters. CDF, cumulative distribution function

To identify potential molecular subtypes of GC, the GC patients were classified into different clusters by consensus clustering (*k* = 2–9) using the “ConsensusClusterPlus” package according to the expression levels of P-CA regulatory factors. The result was visualized in a consensus matrix heatmap (Fig. 2A) when k = 3, the consensus matrix showed the cleanest separation between clusters (Fig. 2B). Then, the three clusters with significantly different clinical outcomes and gene expression patterns were termed cluster A, cluster B, and cluster C. In cluster C, a substantial portion of the P-CA genes was highly expressed, and in contrast, the majority of these genes were expressed at low levels in cluster B (Fig. 2C). In addition, Kaplan–Meier survival curves showed a significant survival difference among the different clusters (Fig. 2D).

### Characteristics of TME cell infiltration and biological behavior under different P-CA clusters

**Fig. 3:**
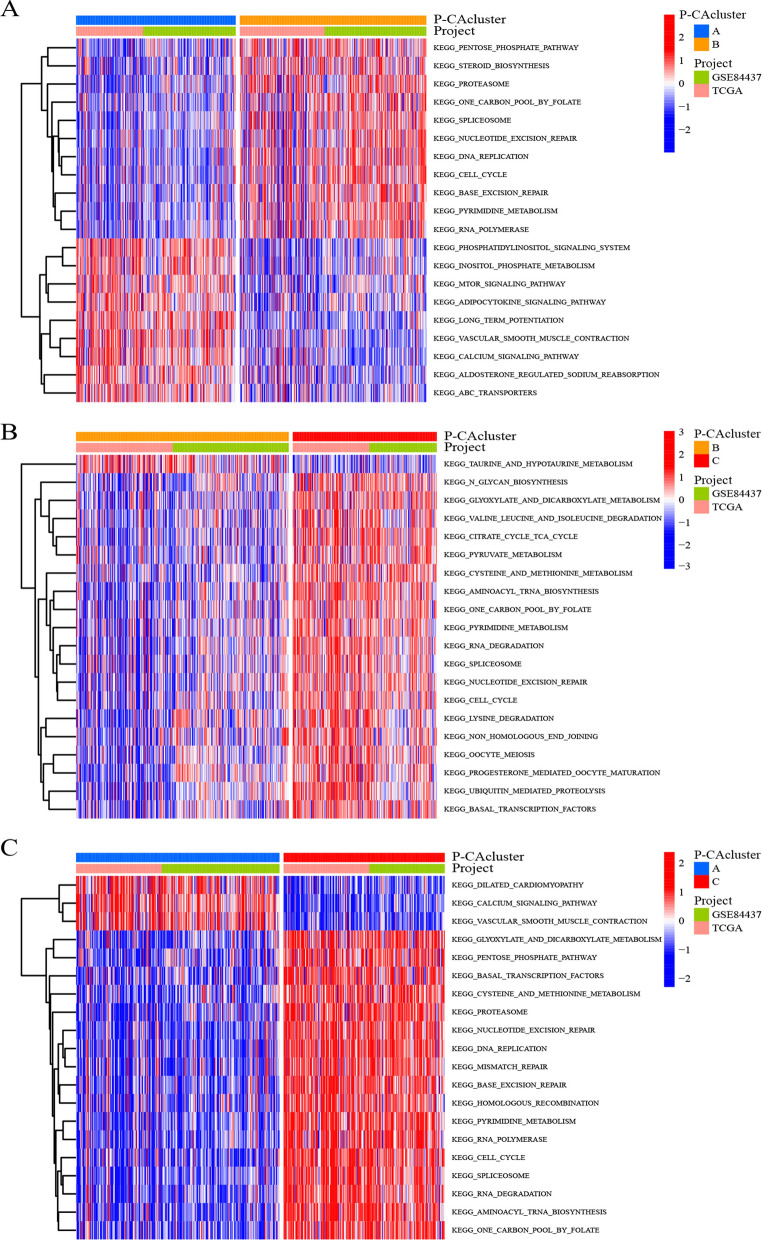
GSVA analysis of different P-CA clusters. Highly enriched KEGG pathways in distinct P-CA clusters as identified in each comparison are visualized by a heat map. **A** P-CA cluster A vs. cluster B. **B** P-CA cluster B vs. cluster C. **C** P-CA cluster A vs. cluster C. GSVA, gene set variation analysis; KEGG, Kyoto Encyclopedia of Genes and Genomes

We further investigated whether biological behaviors differed in different P-CA clusters by the GSVA enrichment analysis. As shown in Fig. 3A, cluster A was predominantly enriched in the pathway of signal transduction and organismal systems, such as the mTOR signaling pathway and vascular smooth muscle contraction. While cluster B was markedly activated in pathways of DNA metabolism processes (transcription, DNA replication, and repair) and metabolism, including spliceosome, nucleotide excision repair, DNA replication, base excision repair, pentose phosphate pathway, steroid biosynthesis, one carbon pool by folate, and so on. Cluster C exhibited a similar trend to cluster B, but it was more enriched in DNA metabolism processes pathways (Fig. 3B and C).

**Fig. 4:**
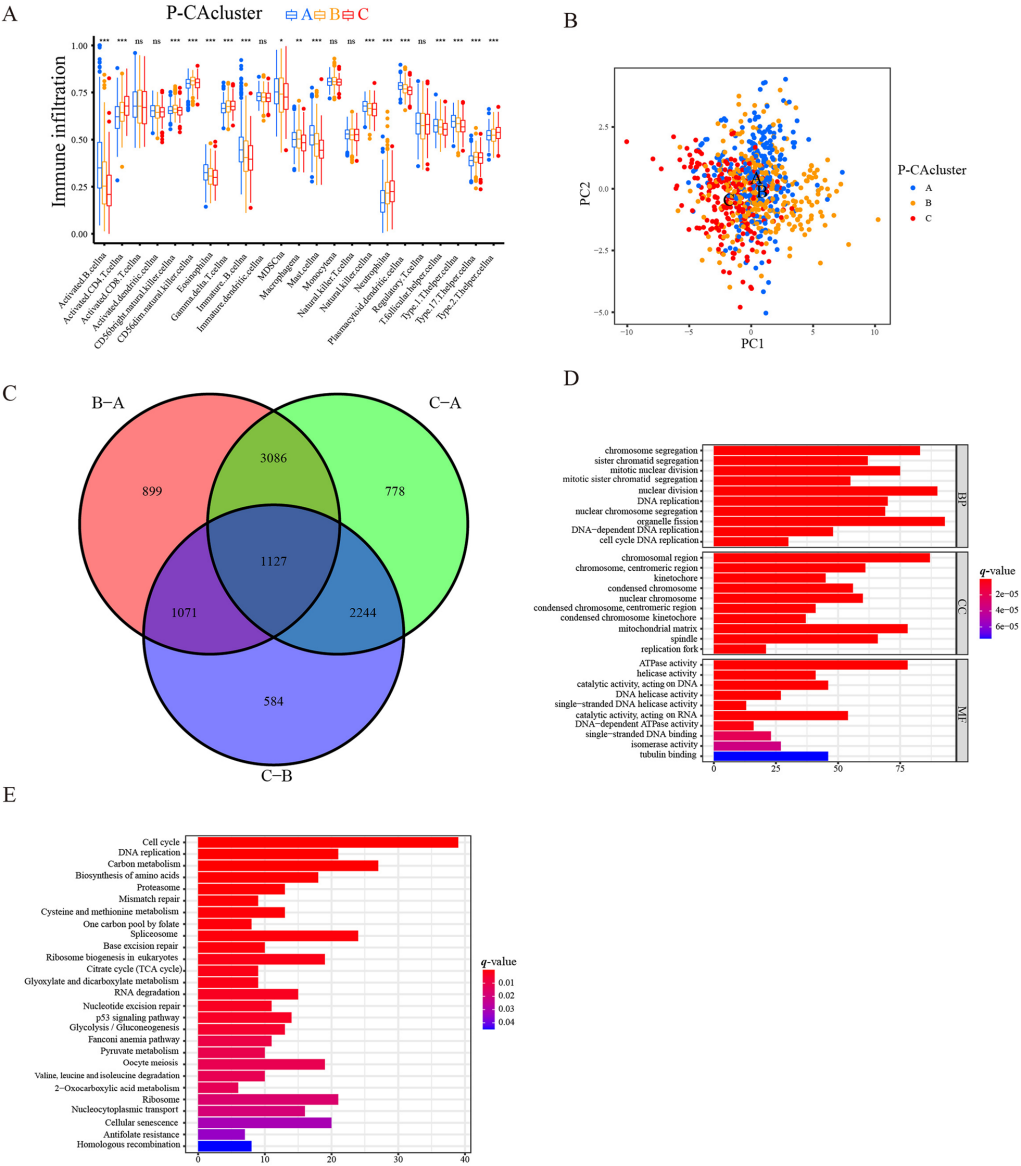
TME immune cell infiltration and transcriptome characterization in three P-CA clusters. **A** Assessing the abundance of infiltrated immune cells among three distinct P-CA clusters. *p < 0.05, **p < 0.01, ***p < 0.001, ns, non-significant, and *p*-value < 0.05 was considered statistically significant. **B** Scatter plot of transcriptome features via Principal component analysis. **C** Venn plots show the number of unique and shared DEGs from different comparisons among three P-CA clusters. **D** GO enrichment analysis of the overlapping DEGs among distinct P-CA clusters. The length and color depth of the bar stands for the number of genes enriched and the *q*-value of each GO term, respectively. **E** KEGG pathway enrichment analysis of overlapping genes. TME, tumor microenvironment; GO, Gene Ontology; KEGG, Kyoto Encyclopedia of Genes and Genomes

Next, we sought to understand whether the P-CA regulators had an impact on GC TME, so the ssGSEA enrichment analysis was conducted to characterize the cell infiltration landscape among three clusters. We noticed that P-CA cluster A has a higher degree of immune cell populations including activated B cell, eosinophil, immature B cell, myeloid-derived suppressor cell (MDSC), mast cell, natural killer cell, plasmacytoid dendritic cell, T follicular helper cell, and T helper cell type 1 infiltration than the two other clusters. Cluster B demonstrated a higher level of CD56 bright natural killer cell and CD56 dim natural killer cell infiltration. Cluster C was highly infiltrated with CD4 + T cells and neutrophils (Fig. 4A). Previous research declared that TME can be divided into three distinct immune subtypes according to the different degrees of infiltration of immune cells and stromal cell [28]. In the present study, P-CA cluster A was characterized by abundant infiltrating cells and MDSCs along with mTOR signaling pathway activation, which can be perceived as the immune-excluded phenotype. High infiltration of CD4^+^ T cells and high DNA damage repair were observed in P-CA cluster C, which was referred to as the inflamed phenotype.

Principal component analysis indicated that the three clusters can be discriminated well by the expression of P-CA regulators (Fig. 4B). 1127 overlapping DEGs among the three clusters were selected according to the pairwise comparisons to further unearth the underlying biological characteristics of each P-CA cluster (Fig. 4C). To further understand the relevant biological functions of the identified P-CA cluster-related DEGs, functional enrichment was performed. For the biological processes, the P-CA cluster-related DEGs mainly participated in cell proliferation processing such as organelle fission, chromosome segregation, mitotic nuclear division, nuclear division. For the cellular component, genes were mainly enriched in the chromosomal region, mitochondrial matrix, spindle, chromosome centromeric region. Furthermore, ATPase activity, catalytic activity acting on DNA and RNA were the principally enriched terms in molecular function (Fig. 4D). Simultaneously, KEGG signaling pathway enrichment analysis was performed to confirm the association of the P-CA cluster-related DEGs with signaling pathways, As shown in Fig. 4E, these genes were major involved in cell cycle, carbon metabolism, spliceosome, and DNA replication. Taken together, the results suggested that there is an association between P-CA cluster-related DEGs and cell division and proliferation.

### Construction of P-CA-related gene signature

**Fig. 5:**
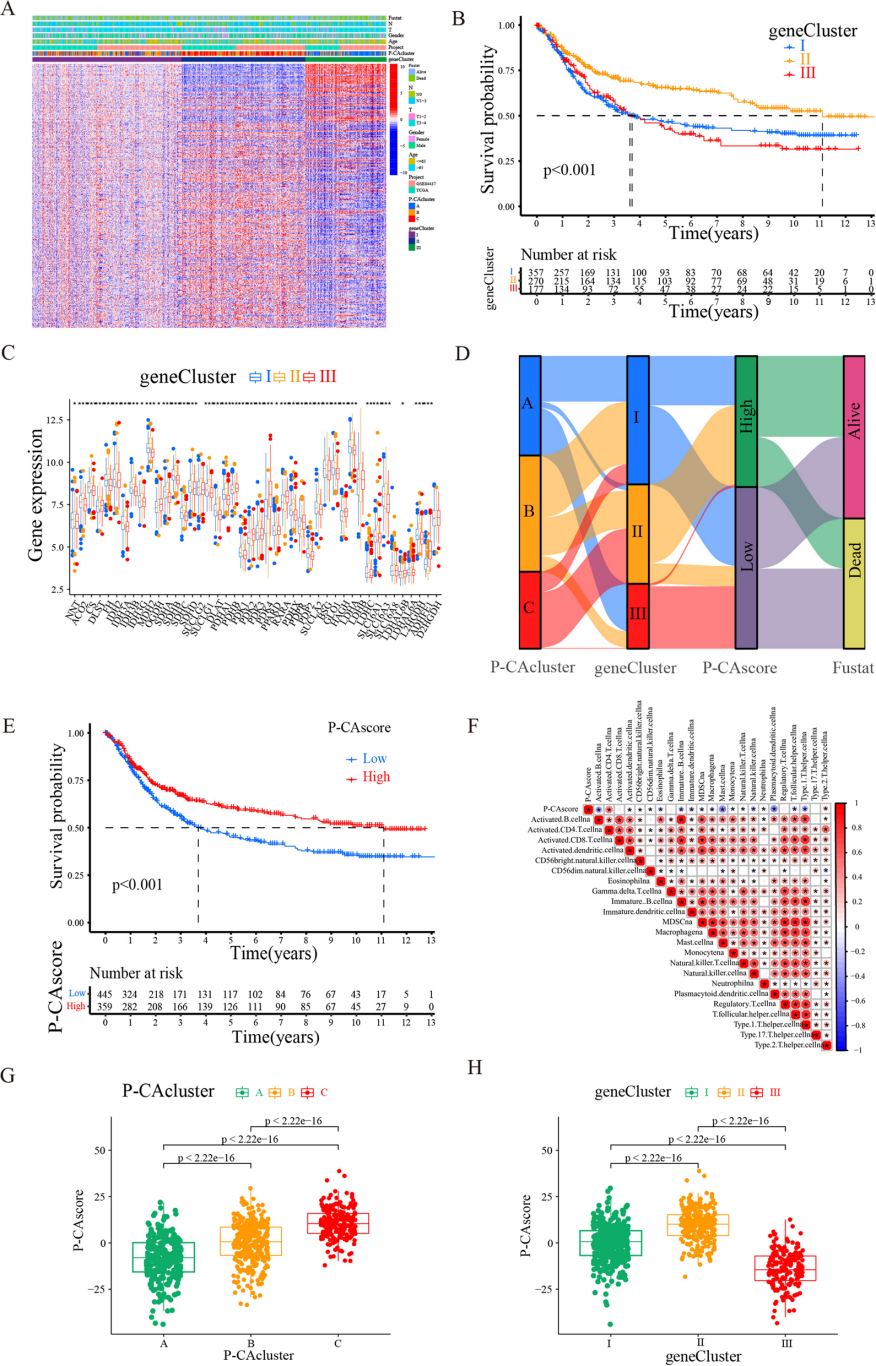
Feature selection and signature construction process. **A** Heatmap for the relationship of clinicopathologic characteristics with P-CA gene clusters. **B** Kaplan-Meier survival curves reveal the significant difference in prognosis advantage among three distinct P-CA gene clusters (log-rank tests, *p*-value < 0.001). **C** The difference in the expression of P-CA-related genes among three distinct P-CA gene clusters. **D** Alluvial diagram showing the relations of P-CA cluster, gene cluster, risk group, and status. **E** Kaplan–Meier curves revealed that the P-CA score was markedly related to the overall survival of patients with gastric cancer (log-rank tests, *p*-value < 0.001). **F** Correlation between P-CA score and multiple immune infiltrating cells. A negative correlation is marked with blue and a positive correlation is marked with red. * was considered statistically significant. **G** Differential expression analysis of the P-CA score in the P-CA cluster. **H** Difference analysis of the P-CA score in the gene cluster

To construct a more accurate gene signature with prognostic significance, the 1127 DEGs were subjected to univariate Cox regression analysis, and prognosis-related genes were subsequently used for further analysis. Clustering results showed a conspicuous difference among the three gene clusters when *k* = 3 (Additional file 2: Fig. S2). A heat map was drawn from differential genes among different gene clusters, which allowed for quick visualization of the clinicopathological differences across gene clusters (Fig. 5A). These prognostic-related genes were the most highly expressed in gene cluster II and showed low expression in gene cluster III. Next, a Kaplan-Meier survival analysis was performed to investigate whether there were significant differences in the survival outcome among the three gene clusters. As shown, patients in gene cluster II had the best survival prognosis, while gene cluster III had the worst one (Fig. 5B). In addition, we observed significant differences in the expression of P-CA regulators among the three gene clusters (Fig. 5C). Considering the individual differences and complexity of P-CA, a scoring system was developed based on P-CA-related gene expression to quantify the P-CA pattern in GC patients. Patients were stratified into two groups according to low or high P-CA scores using the median value. The alluvial diagram elaborated the survival outcomes of the patients in the high and low P-CA score groups from different P-CA clusters and gene clusters. It’s clear that most of the patients in P-CA cluster C lean toward gene cluster II, whereas the majority of patients within gene cluster II flow to the high P-CA score group, which had a better prognosis (Fig. 5D). This was consistent with the aforementioned results that patients in P-CA cluster C and gene cluster II had better survival outcomes. Survival analysis was conducted to identify the prognostic value of the P-CA score, and Kaplan-Meier survival curves showed that for all GC samples, the high P-CA score group had better overall survival than the low P-CA score group (Fig. 5E). Furthermore, immune correlation analysis confirmed the positive association between CD4^+^ T cells and P-CA score (Fig. 5F). Differences in the P-CA score were compared among the three P-CA clusters and gene clusters, and the results showed that P-CA cluster C and gene cluster II exhibited the highest scores, respectively (Fig. 5G and H), which provided good evidence to prove the high accuracy of the P-CA scoring system for survival prognosis judgment.

### Tumor somatic mutation and immune functions in distinct P-CA-score groups

**Fig. 6:**
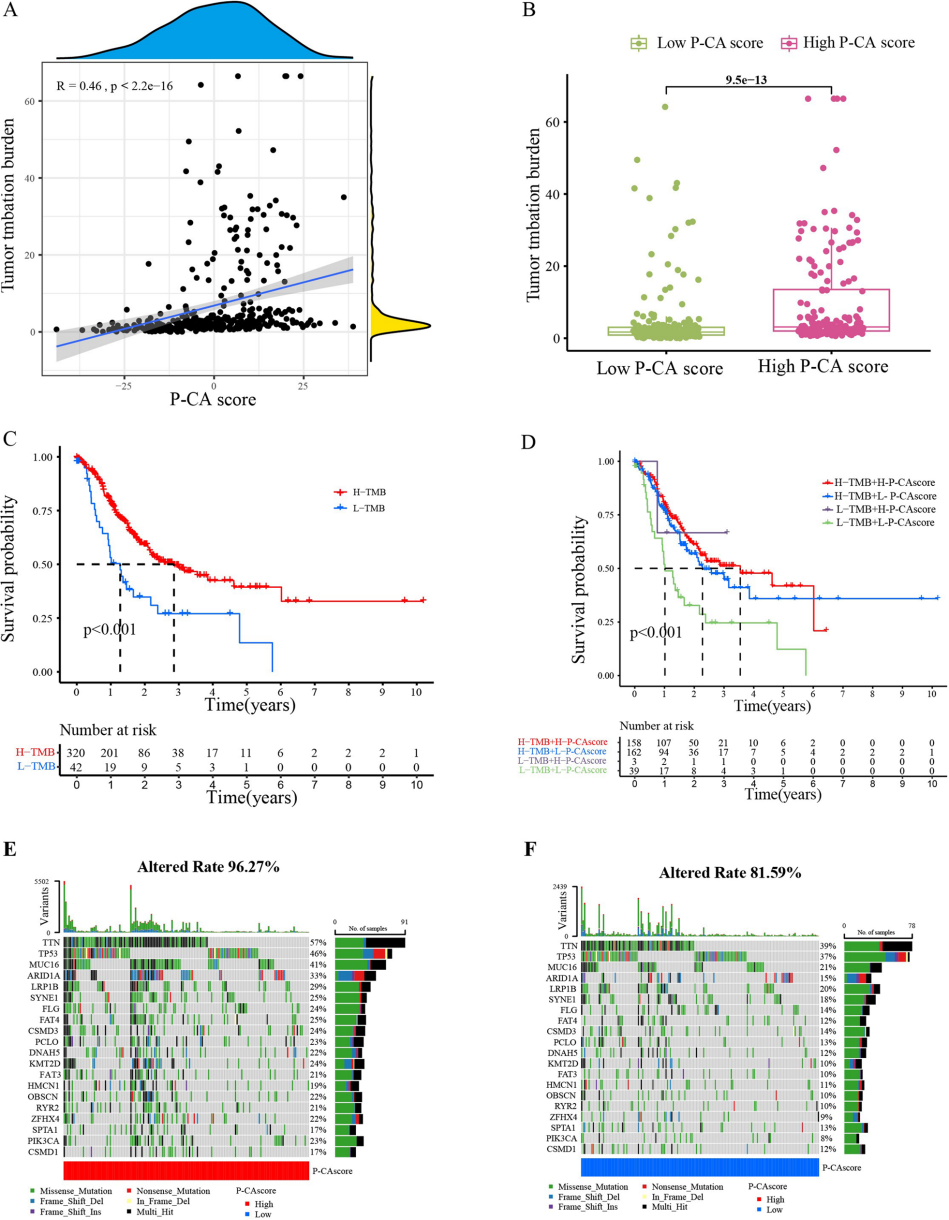
Tumor somatic mutation landscape of GC patients. **A** Dot plots of correlations between P-CA score and TMB. **B** TMB difference between high and low P-CA score groups. **C** Kaplan-Meier survival curve showing the overall survival between the low or high TMB group (log-rank tests, *p*-value < 0.001). **D** Combined survival analysis of TMB with P-CA score (log-rank tests, *p*-value < 0.001). **E**, **F** The somatic mutation features in GC patients with high or low P-CA scores

Tumor mutational burden (TMB) is associated with the efficacy of immunotherapy [29, 30]. Thus, correlation analysis was conducted to assess the associations between P-CA scores and TMB, which indicated that P-CA scores were positively correlated with TMB (Fig. 6A, B). Simultaneously, survival analysis of TMB revealed that the high TMB group indicated a better prognosis for patients (Fig. 6C). Importantly, the survival curves with the integration of TMB and P-CA scores showed that patients in group with high TMB and the high P-CA score showed the best outcomes (Fig. 6D). In addition, the differences in somatic-mutation frequencies between the high and low P-CA groups in GC patients were explored, which demonstrated a higher mutation frequency (96.27%) in the high P-CA group than that in the P-CA-low group (81.59%). The top five mutated genes were *TTN*, TP53, MUC16, ARID1A, and LRP1B in both groups (Fig. 6E and F).

**Fig. 7:**
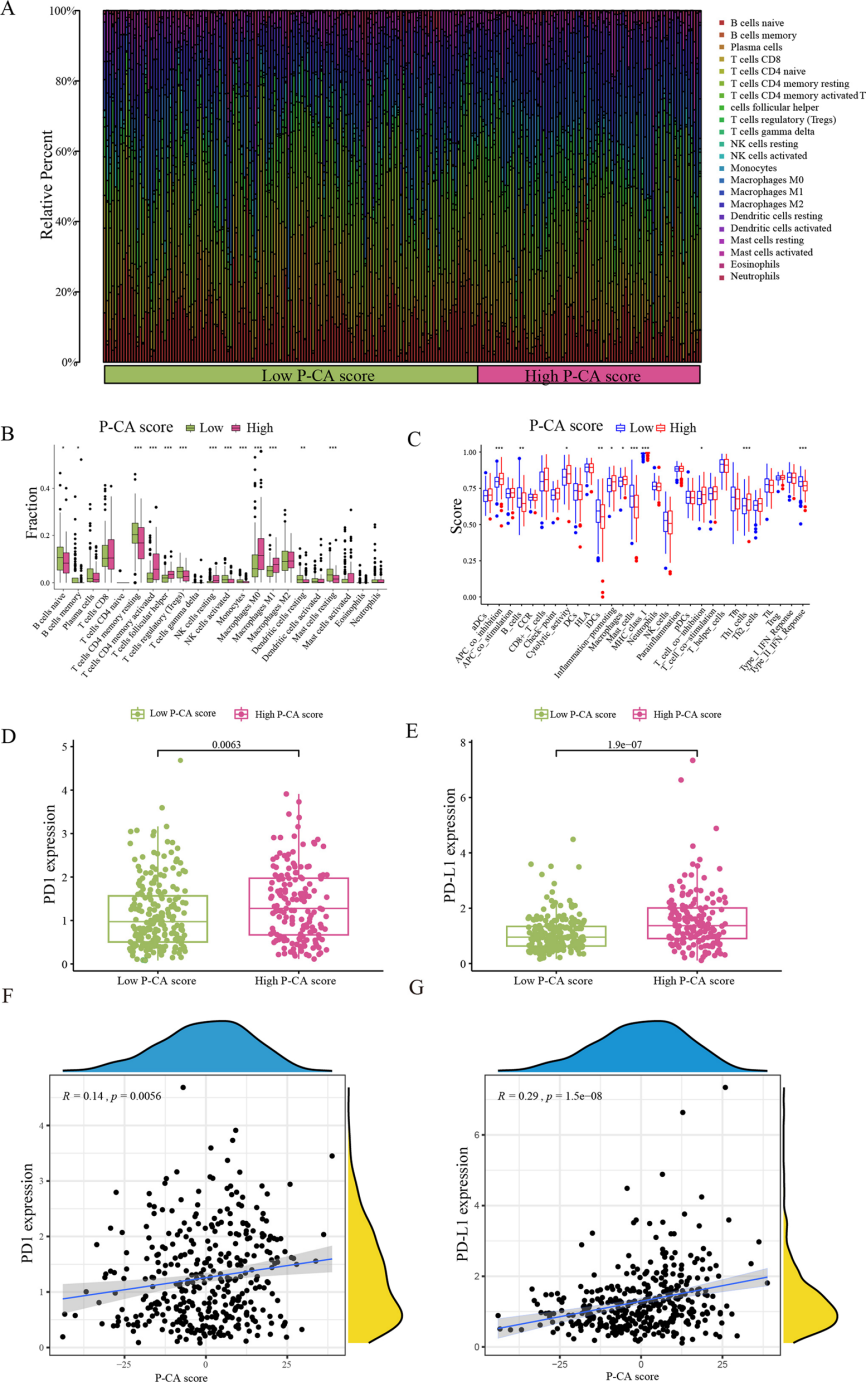
Immune function evaluation between the two P-CA score groups. **A** Immune infiltration status for low and high P-CA score groups. **B**, **C** The box plots show the comparison of immune cells’ abundance and immune-related functions between the two groups. **D**, **E** PD1 and PD-L1 expression levels in two distinct groups. **F**, **G** Correlations between P-CA score with PD-L1 expression or PD-1 expression. The * represents *p*-value < 0.05, ** represents *p*-value < 0.01, *** represents *p*-value < 0.001, and *p*-value < 0.05 was considered statistically significant

Researchers have suggested that alterations in tumor metabolism facilitated the accumulation of acidic metabolites in the TME and promoted tumor cells to escape immune surveillance [31]. Therefore, we contemplated whether the P-CA score affected immune infiltrates. Results showed that the distribution of immune cells was different between the two P-CA score groups. The infiltration density of activated CD4^+^ memory T cells, follicular helper T cells, resting NK cells, M0 macrophages, and pro-inflammatory macrophages (M1) were higher in the high P-CA group, whereas the abundance of naive B cells, T cells CD4 memory resting, and mast cells were elevated in the P-CA-low group (Fig. 7A and B). The immune-related functions in high and low P-CA score groups was recorded in Fig. 7C, and remarkable differences were observed between the two groups in naive B cells, memory B cells, memory resting CD4^+^ T cells, activated CD4 memory T cells, follicular helper T cells, regulatory T cells (Tregs), resting NK cells, activated NK cells, Monocytes, M0 Macrophages, M1 Macrophages, resting dendritic cells, and resting mast cells. The P-CA pattern correlated with the mutation landscape, as well as distinguished TME immune cell infiltration. Currently, immune checkpoint blockade therapies, such as anti-programmed cell death protein 1 (PD1) immunotherapy, have demonstrated considerable clinical benefits in cancer immunotherapy [32]. Programmed cell death protein 1 (PD1) and programmed death-ligand 1 (PD-L1) were measured as important biomarkers for immune checkpoint immunotherapy. Interestingly, we found that the expression levels of PD1 and PD-L1 were elevated in the high P-CA group and positively correlated with the P-CA score (Fig. 7D–G). These findings indicated that patients with GC in the high P-CA score group may benefit more from immunotherapy.

### Immune subtypes and immunotherapy analysis

**Fig. 8:**
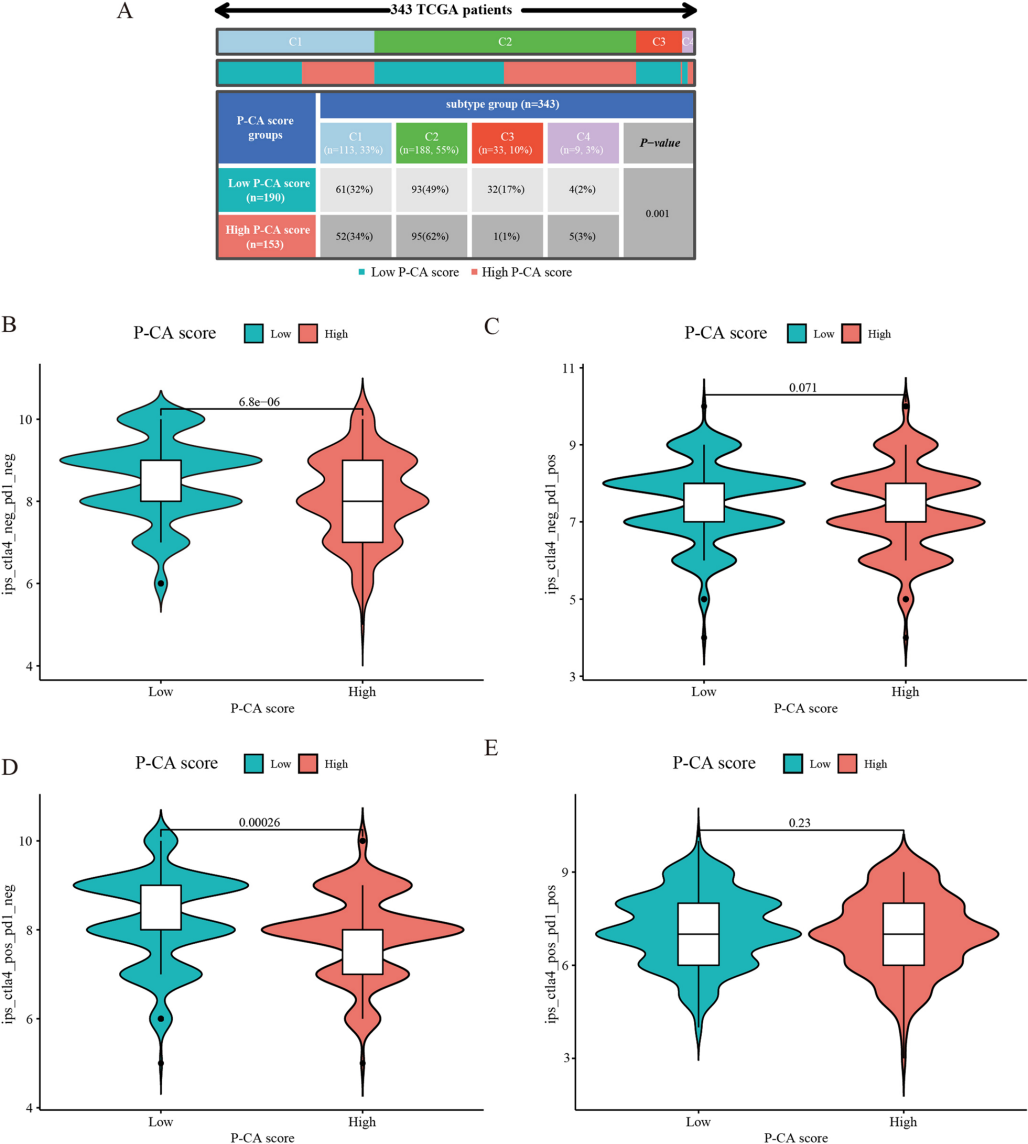
Immune subtypes and immunotherapy analysis of two different P-CA score groups. **A** Distribution of immune subtypes characteristics in GC patients with *p*-value of 0.001. Differential analysis for P-CA-high group and P-CA-low group in **B** CTLA-4 negative and PD-L1 negative therapy, **C** anti-PD-L1 immunotherapy, **D** anti-CTLA-4 immunotherapy, and **E** anti-PD-L1 combined with CTLA-4 immunotherapy. TCGA, The Cancer Genome Atlas

Previously, Thorsson et al. proposed four immune subtypes according to the relative abundance of different subpopulations of immune cells: C1 (wound healing), C2 (interferon-gamma [IFN-γ] dominant), C3 (inflammatory), and C4 (lymphocyte depleted), and the immune subtypes C2 had a high proliferation rate and is highly correlated with mutated gastric cancer [33]. In the present study, GC patients were classified into different immune subtype clusters (C1, C2, C3, and C4). Whether in the high or low P-CA score group, the immune subtype C2 had the largest number of GC patients (Fig. 8A). To investigate the potential role of ICI therapy represented by the CTLA-4/PD-1 inhibitor in the high P-CA and low-P-CA score groups, the immunotherapy scores were analyzed. Differential analysis indicated that the P-CA score had a predictive value for the efficacy of CTLA-4 immunotherapy (Fig. 8B–E).

### MSI analysis

**Fig. 9:**
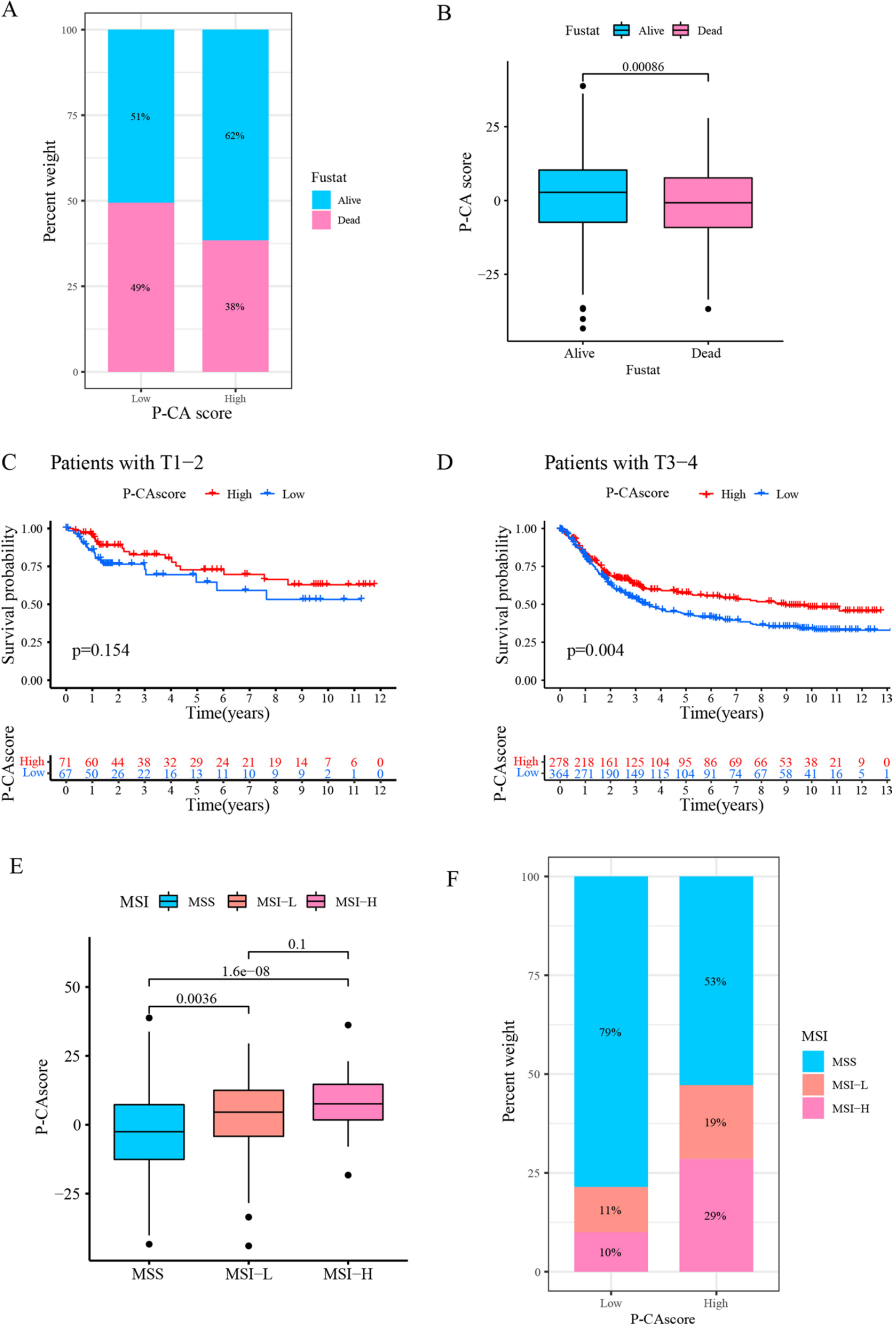
Prognostic value of P-CA score. **A**, **B** The relationship between survival status and P-CA score. **C**, **D** Stratified survival analysis of gastric cancer patients based on P-CA score in T1-2 cohorts and T3-4 cohorts. **E** The relationship between P-CA score and MSI. **F** Analysis of MSI status for gastric cancer patients stratified by P-CA score. MSS, microsatellite stability; MSI-L, microsatellite instability-low; MSI-H, microsatellite instability-high

The survival analysis found that P-CA score was closely related to the survival status, 51% of GC patients showed alive status in the low P-CA group, while 62% of patients were alive in the high P-CA group. This analysis illustrated that patients with high P-CA scores were more likely to be alive (Fig. 9A, B). Stratification analysis using tumor staging revealed that there was a tendency for favorable outcomes in patients with high P-CA scores than those with low P-CA scores in T3–T4 stages (Fig. 9D). Although the difference was not statistically significant in T1–T2 stages, longer survival was observed in the high P-CA score group (*p* = 0.154) (Fig. 9C). A deficiency in mismatch repair (MMR) genes leads to microsatellite instability (MSI), which is classified into MSI-high (MSI-H), MSI-low (MSI-L), and microsatellite stable (MSS) [34]. Considering the greater sensitivity to immunotherapy demonstrated in patients with MSI-H status [35], the relationship between MSI status and the P-CA score was assessed. Most patients with high P-CA scores had MSI-H status (Fig. 9E and F), demonstrating the advantage of immunotherapy in the high P-CA score group.

### Cells administrated with P-CA inhibitors resistant to ferroptotic cell death

**Fig. 10:**
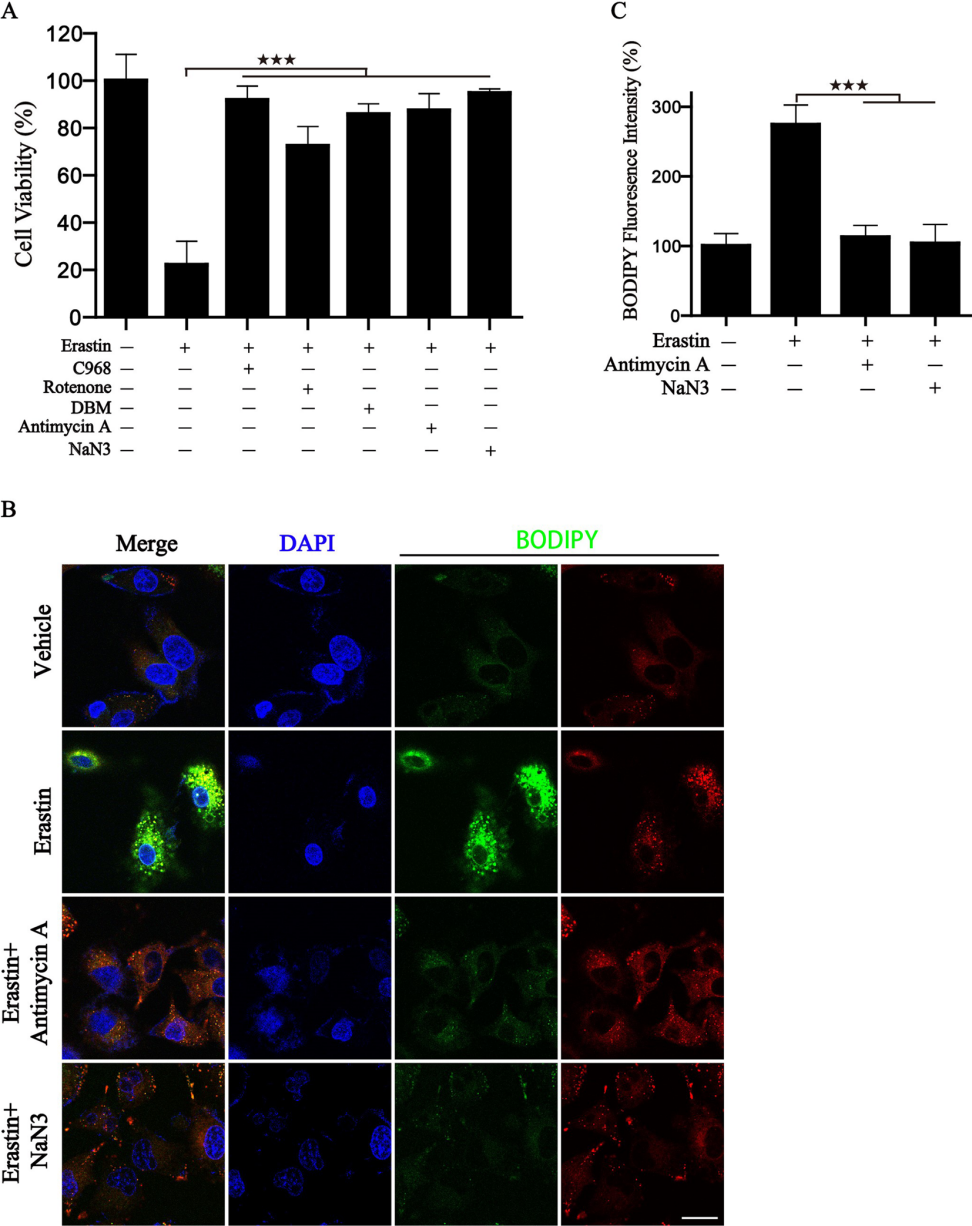
P-CA inhibitors blocked erastin-induced ferroptosis. The BGC823 cells were exposed to erastin in the presence or absence of C968, Rotenone, DBM, Antimycin A, NaN3. then cell viability was measured by CCK8 assay (**A**), and intracellular lipid ROS labeled with BODIPY was detected by confocal laser microscopy (**B**, **C**).

Ferroptosis represents a new programmed cell death that is distinct from apoptosis and necrosis [36, 37]. Our recent studies demonstrated that metabolic reprogramming toward glutaminolysis to fuel the mitochondrial ETC results in oxidative stress, thus predisposing cancer cells to an increased risk of ferroptotic cell death [38]. Hence, achieving ferroptosis via ferroptosis-inducing drugs is emerging as a new alternative therapy modality [39–42]. In the current study, heatmaps of ferroptosis inducers expression among P-CA subtypes and gene clusters were presented. These genes in P-CA cluster C and gene cluster II exhibited their highest expression levels (Additional file 3: Fig. S3A and B). Additionally, the boxplots in Additional file 3: Fig. S3C showed that ferroptosis-related genes were most highly expressed in gene cluster II. Therefore, we can extrapolate that the expression of ferroptosis-inducer genes was positively correlated with the expression of P-CA-related genes. Additionally, we wonder whether the P-CA cluster model could predict the sensitivity to ferroptosis induced therapy. To verify the above conjecture, series of mitochondrial metabolism inhibitors were used in subsequent experiments for the blockade of energy metabolism. As observed from Fig. 10A, cell viability was rescued in BGC823 cells treated with combination therapy along with the addition of mitochondrial respiration inhibitors, compared with erastin alone. Lipid ROS, which serves as a potent ferroptosis marker, was also examined. Similarly, accumulated lipid ROS declined in the combination treatment group (Fig. 10B and C). These experimental results indicated that ferroptotic cell death induced by erastin can be blocked by inhibiting P-CA.

### Hub gene validation

In order to further verify differentially expressed hub genes between GC and normal tissues, we download GEO datasets for validation. The results showed that the expression levels of *LDHA*, *CS*, *IDH3G* were significantly higher in cancer tissues than those in normal tissues (Additional file 4: Fig. S4A–C). Immunohistochemical staining results for *IDH3G* that mined from the HPA further confirmed its high expression in GC tumor tissues (Additional file 4: Fig. S4D).

## Discussion

Metabolic reprogramming is a ubiquitous trait of cancer [43], which allows tumor cells to adapt to a tremendous crisis [44]. The primary metabolic processes modified by tumor metabolic reshaping are glycolysis and the citric acid cycle [45]. Accumulation of intermediate metabolites of the citric acid cycle appears in many types of tumor cells [46], including GC [47, 48]. Increasing evidence has demonstrated the functional role of the accumulated intermediates of P-CA metabolism in cancer progression. However, such metabolites exert antitumor activity under certain conditions because of their cytotoxicity [12], and applying these findings to clinical diagnosis and treatment may have positive effects. The TME is a sophisticated system with different cell types here, including smooth muscle cells, fibroblasts of various phenotypes, granulocytes, myofibroblasts, and immune cells [49]. The interaction among cells in the TME provides a conducive habitat for the survival of tumor cells. The TME can be acidified by high lactate accumulation induced by the enhanced anaerobic glycolytic activity of the tumor, which is considered to actively contribute to tumor immune escape [50]. However, to date, there has been no relevant systematic review on how P-CA alteration impacts TME cell infiltration characteristics in GC and its impact on prognosis. Therefore, the aim of the present study was to develop a P-CA-based scoring system for assessing the effect of P-CA patterns on TME cell infiltration in patients with GC to develop more effective immunotherapeutic strategies.

First, the differential expression of P-CA regulators between normal controls and GC patients was revealed by comprehensively analyzing the multi-omics data of P-CA regulators. Subsequently, three P-CA clusters were established with distinct clinical features and immune infiltration, based on the expression profiles of 49 P-CA regulators. Currently, three distinct immune subtypes were reported: immune-desert, immune-excluded, and inflamed phenotypes [28]. In the present study, P-CA cluster A, with the worst prognosis, presented a TME highly infiltrated by immune cells, along with MDSCs and activation of the mTOR signaling pathway, which was in line with characteristics of the immune-excluded phenotype. Cluster C, with the best prognosis, was characterized by a high infiltration of CD4 + T cells and enrichment in genetic information processing pathways. CD4^+^ T cells are associated with the production of IFN-γ and present tumor antigens to CD8^+^ T cells. Thus cluster C matched the features of inflammatory phenotype. Immune checkpoint inhibitors are more effective in treating inflamed tumors than non-inflamed ones [28]. Therefore, the P-CA cluster was analyzed to improve understanding of the TME.

Cancer immunotherapy is currently a research hotspot and an important adjunct to traditional cancer therapies. Immune checkpoint blockade is a promising modality in clinical immunotherapy by activating the immune system, such as PD-1/PD-L1 blockade and single-agent anti-CTLA-4 [32]. Immunotherapeutic strategies have become popular in GC. However, not all patients can benefit from immunotherapy [51]. Therefore, there is an urgent need to identify molecular biomarkers for predicting treatment benefits and risk stratification during GC treatment. Therefore, 1127 DEGs among the three P-CA clusters were identified and investigated in the subsequent Gene Ontology (GO) enrichment analysis and Kyoto Encyclopedia of Genes and Genomes (KEGG) pathway enrichment analysis, which demonstrated that the P-CA-related genes were enriched mainly in the growth and proliferation of tumor cells. Furthermore, three P-CA-related gene clusters with different clinicopathological characteristics and TME features were identified based on DEGs. To circumvent the impact of individual heterogeneity and specificity, a scoring model was constructed to assess the P-CA pattern in individual patients with GC. P-CA cluster C and gene cluster-II had the highest P-CA scores and showed the best prognosis.

Mutations in tumor cells, which are considered to be non-self-epitopes, can produce neoantigens and improve the efficacy of immune checkpoint inhibitors [52]. A higher TMB suggests a likelihood of a better response to immunotherapy [52]. Furthermore, it has previously been demonstrated that tumors with microsatellite instabilityare associated with a higher TMB, which would be more susceptible to the treatment of immunotherapy [35, 53, 54]. Therefore, further investigations on the relationship between TMB, PD1/PD-L1 expression, and P-CA score were performed. The frequency of total gene mutations in the high P-CA score group was higher than that in the low P-CA score group. The P-CA score was strongly positively correlated with TMB and PD1/PD-L1 expression levels. In our subsequent analysis, the high P-CA score group with significantly higher mutation burdens, higher expression of PD1/PDL1, and microsatellite instability status had a favorable prognosis. These results indicated the effective prognostic value of the P-CA score.

Additionally, the P-CA score can also predict the efficacy of the CTLA-4 immunotherapy. A high P-CA score presented high immune infiltration of CD4^+^ T cells; these immune cells are instrumental for controlling immune function, including immune surveillance of tumor cells [55]. These results indicated that the P-CA score may influence the immunotherapy responses of GC cells, which was in agreement with the findings of previous studies.

Ferroptosis is a novel cell death manner, which has gradually become a promising adjuvant therapeutic measure in cancer treatment since its discovery [56]. Ferroptosis has also been described to be associated with sensitivity to immunotherapy. The mitochondrial electron transport chain and P-CA were responsible for ATP production and biosynthesis of amino and fatty acids. It was reported that P-CA and mitochondrial electron transport play indispensable roles in the execution of ferroptosis [57]. Hence, we sought to reveal whether P-CA pattern can predict the sensitivity of tumor cells to ferroptosis in GC. In this study, the expression of ferroptosis inducer genes was higher in P-CA cluster C and gene cluster II, in which highly expressed P-CA regulators were observed, as well. This recognition suggested that ferroptosis inducers may have high therapeutic indices for high-P-CA-score GC patients. Additionally, results from the experiment proved that inhibition of P-CA and blockade of electron transport could protect tumor cells against the onset of ferroptosis.

Collectively, this study provided insight into immune infiltration correlated with P-CA score, thereby predicting the response of patients with GC to immunotherapy and ferroptosis-based therapy, simultaneously demonstrating the predictive power of P-CA-related genes. The scoring system elucidated in this work could be used for stratifying patients and provided direction for individualized precise therapy.

## Conclusion

In this study, the P-CA patterns were comprehensively assessed based on 49 P-CA regulatory genes. The difference in P-CA patterns may be a significant predictor in immunotherapy by analysis of the diversity and complexity of TME. The higher P-CA score was associated with a better prognosis for GC patients. Additionally, patients with high P-CA scores were found to be more sensitive to ferroptosis inducers. This may have important implications for the understanding of TME infiltration characteristics and lay a groundwork for guiding individualized precise therapy.

## Supplementary Information

**Additional file 1: Figure S1.** The relative abundance of P-CA regulators in *DLAT* wild and *DLAT* mutation groups is presented

**Additional file 2: Figure S2.** Consensus clustering analysis of DEGs in GC. (A) The cumulative distribution function (CDF) curves in consensus cluster analysis for cluster numbers *k* = 2–9. (B) Relative change in the area under the CDF curve from *k* = 2 to *k* = 9. (C) The tracking plot for *k* = 2–9; The vertical axis shows consensus matrix *k*-value, the horizontal axis represents the GC samples. (D-L) Consensus matrix heat map when *k* = 2-9. DEGs differentially expressed genes; CDF, cumulative distribution function.

**Additional file 3: Figure S3.** The correlation of the ferroptosis with P-CA. Heatmap of ferroptosis genes expression among P-CA clusters (A), and gene clusters (B). (C) Differences in the expression of genes related to ferroptosis among the three gene clusters. (The * represents *p*-value < 0.05, ** represents *p*-value < 0.01, *** represents *p*-value < 0.001, and *p*-value < 0.05 was considered statistically significant).

**Additional file 4: Figure S4.** Verification of gene expression levels. Three upregulated genes including *LDHA* (A), *CS* (B), *IDH3G* (C). Immunohistochemical staining of *IDH3G* (D)

## Data Availability

All data in this study can be obtained from the Gene-Expression Omnibus (GEO; https://www.ncbi.nlm.nih.gov/geo/) and The Cancer Genome Atlas (TCGA; https://cancergenome.nih.gov/abouttcga/).
