# Supplementary figures and images for: Characterization of pyruvate metabolism and citric acid cycle patterns predicts response to immunotherapeutic and ferroptosis in gastric cancer
Source: Cancer Cell Int. 2022 Oct 13;22:317. doi: 10.1186/s12935-022-02739-z (PMC9563156; doi:10.1186/s12935-022-02739-z)

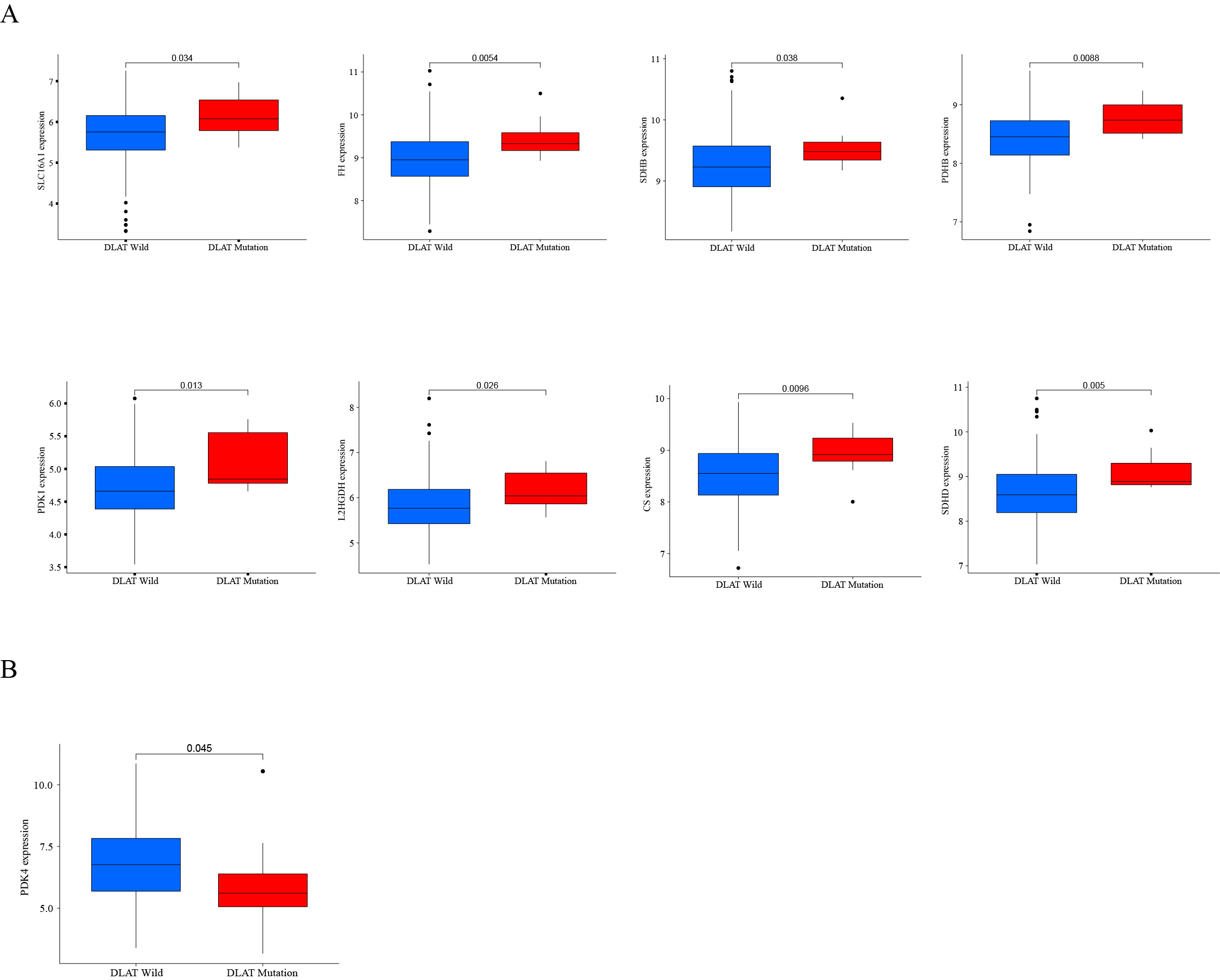

Supplement: Supplementary file 1 — Additional file 1: Figure S1. The relative abundance of P-CA regulators in DLAT wild and DLAT mutation groups is presented [file 12935_2022_2739_MOESM1_ESM.jpg]

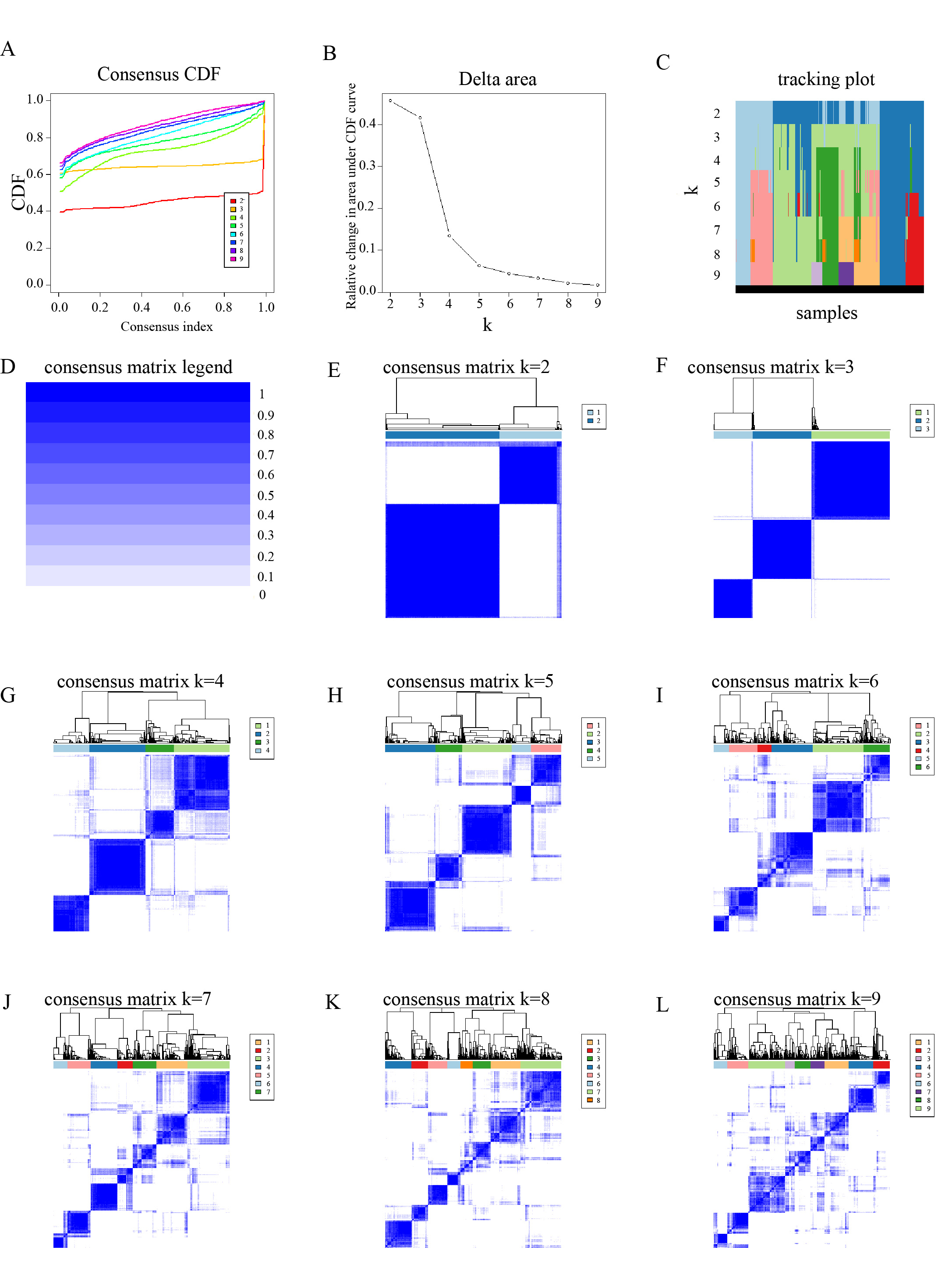

Supplement: Supplementary file 2 — Additional file 2: Figure S2. Consensus clustering analysis of DEGs in GC. (A) The cumulative distribution function (CDF) curves in consensus cluster analysis for cluster numbers k = 2–9. (B) Relative change in the area under the CDF curve from k = 2 to k = 9. (C) The tracking plot for k = 2–9; The vertical axis shows consensus matrix k-value, the horizontal axis represents the GC samples. (D-L) Consensus matrix heat map when k = 2-9. DEGs differentially expressed genes; CDF, cumulative distribution function. [file 12935_2022_2739_MOESM2_ESM.jpg]

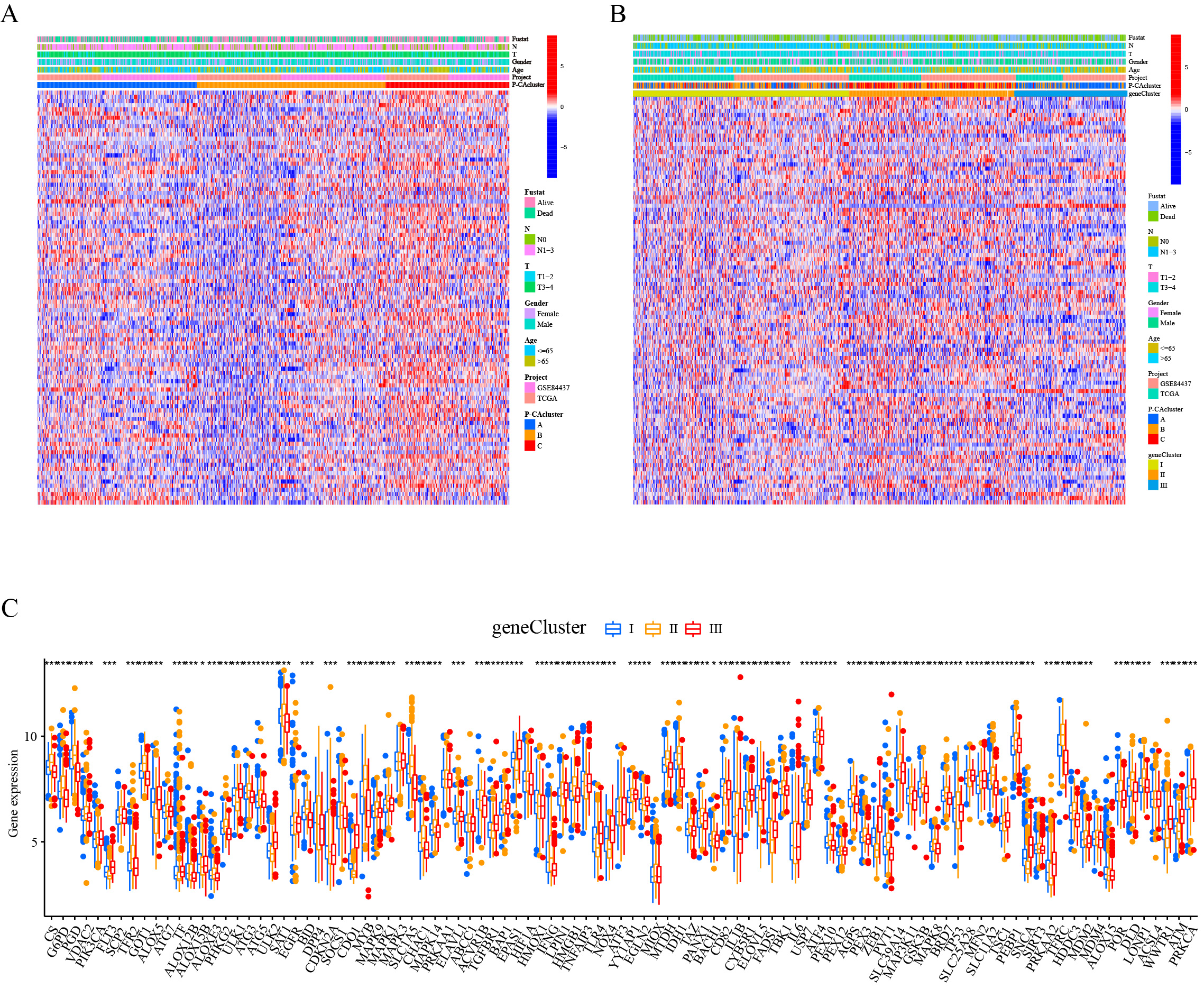

Supplement: Supplementary file 3 — Additional file 3: Figure S3. The correlation of the ferroptosis with P-CA. Heatmap of ferroptosis genes expression among P-CA clusters (A), and gene clusters (B). (C) Differences in the expression of genes related to ferroptosis among the three gene clusters. (The * represents p-value < 0.05, ** represents p-value < 0.01, *** represents p-value < 0.001, and p-value < 0.05 was considered statistically significant). [file 12935_2022_2739_MOESM3_ESM.jpg]

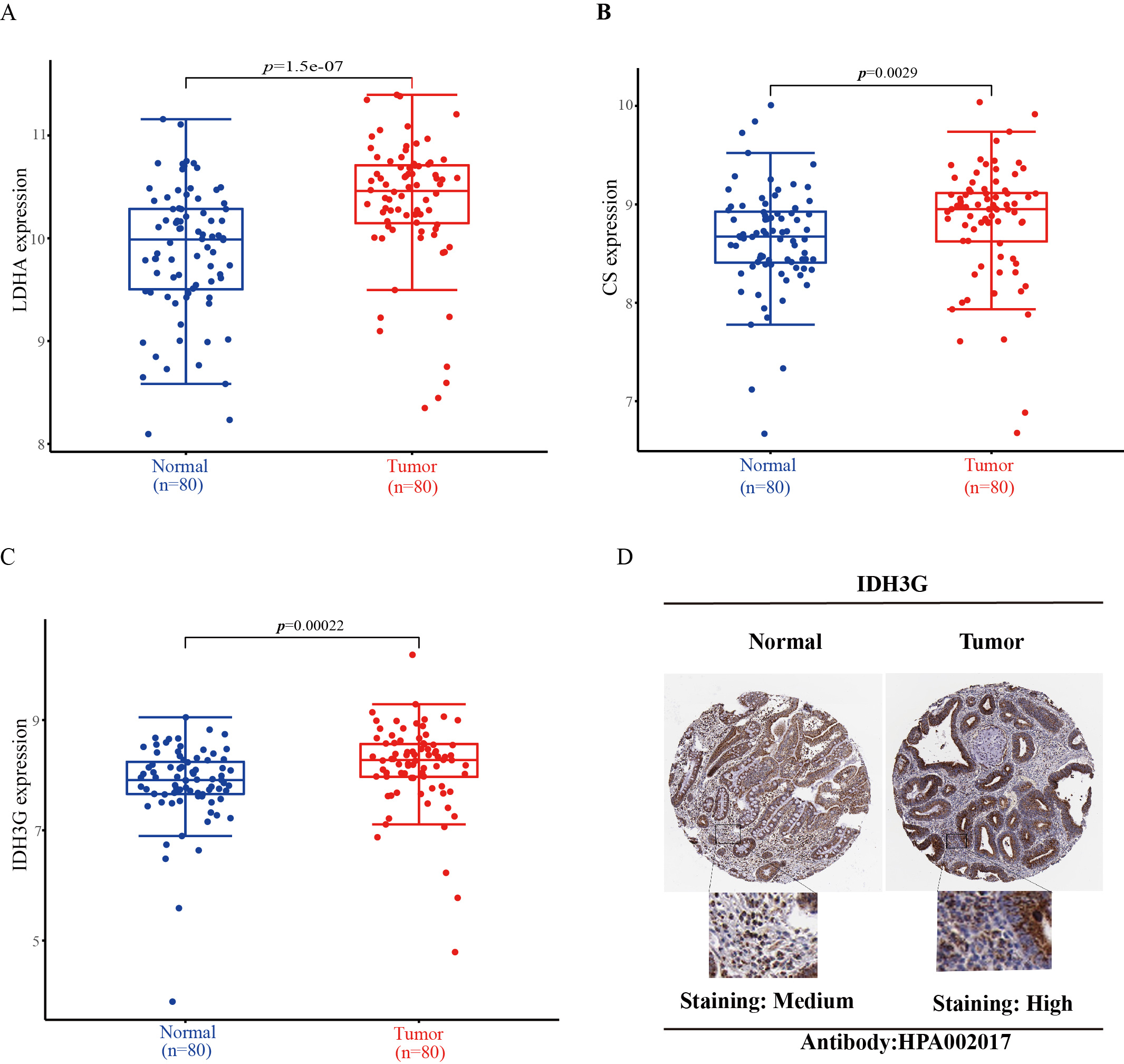

Supplement: Supplementary file 4 — Additional file 4: Figure S4. Verification of gene expression levels. Three upregulated genes including LDHA (A), CS (B), IDH3G (C). Immunohistochemical staining of IDH3G (D) [file 12935_2022_2739_MOESM4_ESM.jpg]
